# The need for fast-track, high-quality and low-cost studies about the role of the BCG vaccine in the fight against COVID-19

**DOI:** 10.1186/s12931-020-01439-4

**Published:** 2020-07-11

**Authors:** Marcos Pereira, Enny Paixão, Anete Trajman, Ramon Andrade de Souza, Marcio Santos da Natividade, Julia M. Pescarini, Susan Martins Pereira, Florisneide Rodrigues Barreto, Ricardo Ximenes, Margareth Dalcomo, Maria Yury Ichihara, Ceuci Nunes, Manoel Barral-Netto, Maurício L. Barreto

**Affiliations:** 1 Institute of Collective Health, Federal University of Bahia, Salvador, Brazil; 2 Center for Data and Knowledge Integration for Health (CIDACS), Fiocruz, Salvador, Brazil; 3 Faculty of Epidemiology, London School of Hygiene and Tropical Medicine, London, UK; 4 Instituto de Medicina Social, Universidade do Estado do Rio de Janeiro, Rio de Janeiro, Brazil; 5 Departamento de Medicina Tropical, Universidade Federal de Pernambuco, Recife, Brazil; 6 Centro de Referência Hélio Fraga, Fundação Oswaldo Cruz, Rio de Janeiro, Brazil; 7 Hospital Couto Maia, Secretaria Estadual de Saúde da Bahia, Salvador, Brazil; 8 Instituto Gonçalo Moniz - Fundação Oswaldo Cruz (Fiocruz), Salvador, Brazil; 9 Faculdade de Medicina, Universidade Federal da Bahia, Salvador, Brazil

## Abstract

Bacillus Calmette-Guérin (BCG) vaccination is routine and near-universal in many low- and middle-income countries (LMIC). It has been suggested that BCG can have a protective effect on COVID-19 morbidity and mortality. This commentary discusses the limitations of the evidence around BCG and COVID-19. We argue that higher-quality evidence is necessary to understand the protective effect of the BCG vaccine from existing, secondary data, while we await results from clinical trials currently conducted in different settings.

## Letter to the editor

Neonatal *Bacillus Calmette-Guérin* (BCG) vaccination is routine and near-universal in many low- and middle-income countries (LMIC). By the end of March 2020, during the unpreceded global health crisis generated by the COVID-19 pandemic, a few ecological studies suggested a protective effect of the BCG vaccine, with less morbidity and lower mortality from the severe acute respiratory syndrome coronavirus-2 (SARS-CoV-2) [1–4]. These studies have drawn attention to this century-old vaccine and a few clinical trials are underway to repurpose BCG vaccination.

According to these studies, the incidence of COVID-19 is four times higher, with a 21-fold increase in death rates in high- and middle-income countries (HMIC) without universal BCG vaccination when compared to those with this policy [1]. The earlier the implementation of the policy in the country, the lower the number of COVID-19 related deaths, possibly due to a larger proportion of vaccinated older adults [1, 2]. However, those studies included countries that were still in early phases of the epidemic back then, including Brazil, which now accounts with the highest number of daily COVID-19 deaths. Also, the observed association was not sustained in countries with high COVID-19 testing rates [3, 4].

The potential protective effect of neonatal BCG vaccination on COVID-19 severity was suggested due to the lower COVID-19 mortality in some European and Asian countries (China, South Corea, India, Russia and Japan), where the vaccination is mandatory [5, 6]. Additionally, COVID-19 mortality is higher where BCG vaccination has been discontinued for more than 20 years [7]. France, for instance, registered lower COVID-19 mortality than Italy, where the vaccination is no longer mandatory [5–8]. However, there is conflicting data on the protective effect of BCG vaccination. In Italy, BCG vaccination was interrupted in 2001 and COVID-19 mortality rates were high among elders [5–8]. In the UK, where BCG vaccination was recently restarted for TB high-risk groups such as migrants and individuals living in social deprived conditions, COVID-19 mortality rates were higher in these groups than in the overall population [5–8]. Finally, countries using the original BCG strains registered lower COVID-19 case fatality rate if compared to European countries that are using modified BCG strains (Japan - 5.4%; Brazil- 4.7% and Rússia-1.4% vs France-15.1%, Italy- 14.5% and United Kingdom 14.0%) [5–8], suggesting that different BCG strains can have different effects on COVID-19 severity and mortality. Regardless of the controversial findings, ecological bias and the large numbers of potential confounders, such as age, socio-economic factors, health system capacity, stage of the epidemic and the proportion of the population tested, among others, hamper the confiability of these ecological observations and the inference to the individual level.

The alleged protective effect could be the result of trained immunity stimulation from BCG. BCG vaccination can reduce viremia from other respiratory tract viral infections [9, 10]. This would be achieved by boosting non-specific Th1 and Th17 responses for at least one year after vaccination [9], inducing trained immunity, the equivalent of immunological memory for the innate immune response. BCG vaccination followed by a challenge with attenuated Yellow Fever Virus leads to macrophage reprogramming, with a central role of interleukin 1 beta (Il-1b), a hallmark of trained immunity [9]. Trained immunity results in a long-lasting functional programming of innate immune cells that, even in a quiescent state, can be recovered by endogenous or exogenous stimuli resulting in a better-regulated response against a new challenge [11].

In the absence of a specific treatment and vaccine for COVID-19, even a small protective effect of a cheap and safe vaccine such as BCG could be a valuable tool for fighting the disease. If this effect can be confirmed by higher-quality evidence, BCG vaccine could be used to improve clinical outcomes of the most vulnerable, reducing the need for hospital beds, ventilators, and the overall impact on LMIC countries’ fragile health systems. Additionally, applied to healthcare professionals, it could protect the healthcare force, reducing the burden upon them, and the absenteeism from the disease.

High-income countries without BCG vaccination recommendations are planning clinical trials to test the protective effect of BCG on COVID-19 among healthcare workers and older adults [12, 13]. To support the rationale of these trials, LMIC such as Brazil, where BCG vaccination has been used since 1929 - with universal coverage since 1973, are strategic to provide timely evidence on this potential effect of the BCG vaccine, by conducting large case-control studies, which are usually faster and less expensive than clinical trials. Our group is currently using Brazilian routine programmatic data from the existing information systems to analyse the effect of neonatal BCG immunization on COVID-19 clinical outcomes. In addition, data from a large randomized clinical trial (*REVAC-BCG)*, conducted in over 350 thousand school-aged children in two large Brazilian cities in 1996–1997 to investigate the effect of BCG vaccination and revaccination on tuberculosis is being linked to the COVID-19 database to provide more insights on this matter [14]. As the BCG revaccination was ineffective for protection against tuberculosis, the control arm was not revaccinated; thus, the randomization carried out in the past is still valid over the years.

Producing high-quality evidence of the effect of BCG vaccination on COVID-19 outcomes is essential for decision-makers to eventually recommend the BCG vaccination as a possible intervention for COVID-19. We urge colleagues from LMIC where BCG vaccination policies are in course to produce fast, trustable, and low-cost evidence about the effect of the BCG vaccine on the COVID-19 pandemic, while trials are underway. In case the results are positive, it would summ up with the results of the trials in HMIC in course to speed the decisions on the best rationale for using BCG in the current and future possible waves of the pandemics.

## Data Availability

Not applicable.

## Abbreviations

BCG: *Bacillus Calmette-Guérin*
HMIC: High- and middle-income countries
Il-1b: Interleukin 1 beta
LMIC: Low- and middle-income countries
SARS-CoV-2: Severe acute respiratory syndrome coronavirus 2
REVAC-BCG: Brazilian rendomized trial on revaccination of pre-high school children for protection againt tuberculosis
